# Lambda bacteriophage nanoparticles displaying GP2, a HER2/neu derived peptide, induce prophylactic and therapeutic activities against TUBO tumor model in mice

**DOI:** 10.1038/s41598-018-38371-z

**Published:** 2019-02-18

**Authors:** Atefeh Razazan, Jessica Nicastro, Roderick Slavcev, Nastaran Barati, Atefeh Arab, Fatemeh Mosaffa, Mahmoud Reza Jaafari, Javad Behravan

**Affiliations:** 1 Biotechnology Research Center, Pharmaceutical Technology Institute, Mashhad University of Medical Sciences, Mashhad, Iran; 2 School of Pharmacy, University of Waterloo, Waterloo, Canada; 3 Waterloo Institute of Nanotechnology, University of Waterloo, Waterloo, Canada; 4 Mediphage Bioceuticals, Inc., MaRS Centre, West Tower, Toronto, Canada; 5 Theraphage Inc., Waterloo, Ontario, Canada; 6 Center for Bioengineering and Biotechnology, University of Waterloo, Waterloo, Canada; 7 Nanotechnology Research Center, Pharmaceutical Technology Institute, Mashhad University of Medical Sciences, Mashhad, Iran

## Abstract

Generating a protective and long-lasting immune response is the primary goal in the expanding field of immunotherapeutic research. In current study we designed an immunogenic bacteriophage- based vaccine to induce a cytotoxic T lymphocyte activity against a mice tumor model over-expressing HER2/neu. Bacteriophage λ displaying a HER2/neu derived peptide GP2 was constructed and used as an anti-cancer vaccine in a BALB/c mouse xenograft tumor model. The results of our study indicated that phage nanoparticles displaying GP2 as a fused peptide to the gpD phage capsid protein induced a robust CTL response. Furthermore, the chimeric phage nanoparticles protected mice against HER2/neu-positive tumor challenge in both prophylactic and therapeutic settings. In conclusion, we propose that λ phage nanoparticles decorated with GP2 peptide merit further investigation for the development of peptide-based vaccines against HER2/neu overexpressing tumors.

## Introduction

HER2/neu is a proto-oncogene that is overexpressed in 20–35% of human breast cancers^1^. The protein belongs to the human epidermal growth factor receptor (EGFR) family and is able to elicit cellular and humoral immune responses in patients with breast and ovarian cancers^2^. Immunogenic tumor cells produce multiple tumor-associated antigens (TAAs)^3–5^. There is evidence that self-acting antitumor responses to TAAs may be repelled by the host’s own immune system^6^. Vaccines are designed to incite the intrinsic antitumor immune response by effectively presenting the immunodominant TAAs and to stimulate a potent cytotoxic lymphocyte (CTL) immune response^7^.

GP2 is a highly immunogenic peptide of interest against HER2/neu overexpressing breast cancers^8^. This short peptide is derived from the HER-2/neu protein trans membrane domain (654–662: IISAVVGIL) and is recognized by the endogenous immune system via MHC class I^9^. Safe and efficient HER2-specific immune responses were demonstrated in phase I and II clinical trials with GP2^10^, including a CD8+ cytotoxic T-lymphocyte (CTL) response^11^. GP2 is considered a suitable molecue for peptide vaccine trials and is capable of producing strong immunogenicity^12^.

Particular advantages of peptide vaccines over other conventional vaccines include their safety profile, and the simplicity of their production^7^. To produce a peptide vaccine, the carrier of the immunogenic peptide, e.g. bacteriophage or liposome, plays a crucial role as it should be able to efficiently present target antigens to the immune system^13,14^. In 1988, the first use of phage particles to induce an immune response against the displayed foreign peptides was reported^15^. This is particularly advantageous when targeting self-antigens such as HER2 that mediate key biological functions in the body, as immune responses elicited by whole protein vaccines can stimulate the growth of tumor cells if the antibodies mimic the activity of growth factor ligands^16^.

Some of the reported advantages of bacteriophages include their high stability under a variety of harsh environmental conditions, feasibility of their large-scale production, their non-pathogenic nature, and their inherent biological safety profile in mammalian organisms^17^. Antigen-presenting cells (APCs) readily take and process the immunogenic molecule from the displaying phage. This antigen specific targeting makes phages suitable delivery vehicles for immunization^18^.

Hayes *et al*. (2010) demonstrated that phage vaccines were able to induce both cellular and humoral immune responses^19^. Moreover, Sartorius *et al*. (2008) reported that the filamentous bacteriophage fd displaying the HLA-A2-restricted peptides MAGE-A10 or MAGE-A3 could induce strong antitumor CTL responses both *in vitro* and *in vivo*^20^. The filamentous phage fd displaying RT2 peptide (derived from the reverse transcriptase of HIV-1) was also shown to induce an efficient and specific CTL response against HIV-RT2 in human cell lines and in HLA-A2 transgenic mice^21^. Phage particles are inherently immunogenic and can serve as effective natural adjuvants. As such, phage display vaccines negate the need for adjuvants that are frequently used along with recombinant proteins and synthetic peptides to improve immune response^22^.

Bacteriophage lambda (λ) is a temperate phage characterized by a double-stranded DNA genome of 48,502 bp^23^. It exclusively infects *Escherichia coli* (*E. coli*) and can either lysogenize or grow negatively on its bacterial host, although lytic strains exist that are incapable of forming a stable lysogen^24^. Phage λ is preferable to other bacteriophages for protein or peptide display. It has been illustrated to stably display fusion proteins or peptides larger than a few amino acids on its capsid, with copies per virion that are of two to three orders of magnitude higher than filamentous phage display vectors^25^. The capsid of λ is made up of the two major proteins, gpE and gpD^26^. Mikawa *et al*. (1996) determined that both the N and C termini of gpD are neither at the trimer interaction interface nor do they interact with the other major capsid protein gpE and that the terminal tolerance and capacity depends on the peptide or protein that is fused^27^. Subsequent studies demonstrated that gpD was tolerant of peptide and protein genetic fusions at either its N- or C-termini without interfering with λ phage production^28^. The λF7 phage has a mutation in gpD gene which results in a truncated gpD fragment when translated in a wild type (non-suppressor) host. Through this mutation, a functional gpD protein can be produced when the phage infects amber suppressor strains of *E. coli* or when functional *D* is expressed *in trans*.

In this study, an amber suppressor strain of *E. coli* was used for cloning of a cassette containing the capsid protein (gpD) -linker-polypeptide (GP2). λF7 (λDam15) phage was propagated in an amber suppressor strain where the D-fusion protein, gpD::GP2, was provided *in trans* from a multi-copy temperature-inducible expression plasmid^29^ that produced gpD::GP2 to complement for the *Dam15* mutation of λ and decorate viable phage progeny that can produce plaque in *E. coli* strain BB4. Whereas the λF7 phage has a mutation in gpD gene, it cannot produce phage plating and amplification in BB4. We then assessed both prophylactic and therapeutic administration of the GP2 displaying λ phage nanoparticles in a TUBO tumor model of BALB/c mice.

## Materials and Methods

### Bacterium and phage strains

For phage plating and amplification we used *E. coli* strain BB4 (*supF58 supE44 HsdR514 galK2 galT22 trpR55 metB1 tonA* DE*(lac) U169*)^30^. For construction of the λ vector the phage λF7 *(λimm21Dam15)* was used^23^. The plasmid pGPD, as a general purpose vector was used for cloning and expression of gpD fusion peptide. To produce the fusion peptide, the terminal stop codon from gpD was removed and an in-frame fusion with the GP2 sequence (sense: 5′ATTATTAGCGCGGTGGTGGGCATTCTGTAG 3′) and (anti-sense 3′TAATAATCGCGCCACCACCCGTAAGACATC 5′) was created.

The two fragments were separated by an in-frame short linker (ACTAGCGGGTTCTGGTTCCGGTTCTGGTTCCGGTTCTGGC) that was placed between and followed by a *Kpn*I cut site to maximize fusion functionality and also allow for additional fusions to be designed in the future. The gpD::GP2 sequence was then amplified and cloned into the *Hpa*I and *Nco*I sites on pGPD, placing it under the control of the *P*_*L*_ strong promoter that is regulated by the temperature-inducible λ repressor CI857 that confers temperature-regulated expression^29^.

### Phage amplification and purification

Cultures of transformed Sup^+^ (*SupE*) (pGPD::GP2) *E. coli* cells were grown on plates at 37 °C overnight. Dilutions of primary lysates (1:1000) were prepared in 10 µL of TN buffer (0.01 M Tris–HCl and 0.1 M NaCl, pH 7.8), (Fisher Scientific, USA). Lysate dilutions were added to 700 µL of cells (1 × 10^8^ CFU/mL), incubated for 2 h at experimental room temperature prior to adding 5 ml of top LB agar (LB broth +0.7% agar, Bacto Agar from Difco Laboratories, Sparks, MD) and plates were incubated overnight at 37 °C. Plate lysates were then prepared by adding 10 mL of ice cold TN buffer to the surface of the plate, incubating overnight at 4 °C, then transferring the solution and top agar to a conical tube, mixed and centrifuged at 8,000 RPM (Hettich, Germany) at 4 °C for 20 min. The supernatant was poured into a fresh ice-cold (0 °C) conical tube and 2 µL of chloroform was added. Lysates were then precipitated by centrifuging at 8000 RPM at 4 °C for 10 min. The supernatant was removed and transferred into a new sterile tube. Then 1 µL DNase (Sina Colon, IRAN) was added to the lysate to remove any remaining free DNA in the lysate. The lysates were then passed through a 0.45 μm syringe filter (BD Discardit, India) and kept at 4 °C for further experiments.

For phage purification polyethylene glycol (PEG)−8000 (Fisher Scientific, USA) was added to a final concentration of 10% (w/v). The bacteriophage particles were then recovered by centrifuging at 8000 RPM (Hettich, Germany) at 4 °C for 10 min. The supernatant was discarded and 1 ml TN buffer was added to the pellet and kept at 4 °C overnight. To separate PEG and cell debris from the phage nanoparticles, an equal volume of CHCl_3_ was added. The mixture was vortexed gently for 30 seconds and spun at 4300 RPM (Hettich, Germany) at 4 °C for 15 min. The aqueous phase, which contained the bacteriophage particles, was then removed. The solution was filtered through a sterile 0.45 μm syringe filter (BD Discardit, India). To remove endotoxin (LPS), 1% Triton X-114 was added and the solution was shaken at 4 °C for 30 min (Innova 4080 Incubator shaker). Then the solution was incubated at 37 °C for 10 min and spun 14000 RPM (Hettich, Germany) at 25 °C for 10 min. To completely eliminate endotoxin the phage purification procedure repeated three times. The phage solutions were tittered at each step of purification by standard viability assays on fresh Sup^+^ BB4 (*supE, supF*) *E. coli* cells. Samples were stored at 4 °C.

### Animals and cell lines

All experimental procedures involving animal studies were approved by the Ethical Committee of the Research and Technology Council (RTC) of Mashhad University of Medical Sciences (MUMS) based on animal rights guideline (Education Office, proposal number 98623). Female BALB/c mice (4–6 week-old) were obtained from Iran Pasteur Institute (IPI, Tehran-Iran). The rHER2/neu overexpressing cell line, TUBO, was generously provided by Professor Pier Luigi Lollini (The University of Turin, Orbassano, Italy). The cells were cultured in Dulbecco’s Modified Eagle’s Medium (DMEM) supplemented with FBS (fetal bovine serum, 20%). A rHER2/neu negative murine colon carcinoma cell line (CT26) was obtained from the IPI, cultured in RPMI-1640 medium supplemented with FBS (10%), and served as HER2/neu negative controls.

### BALB/c mice immunization

The mice were divided into 3 groups (10 mice in each group). GP2 displaying phages (10^8^ PFU in100 μL per mouse) were injected through subcutaneous route (SC) for three times at consecutive two-week intervals. The controls included λF7 (10^8^ PFU in 100 μL) or 100 µL TN buffer (per mouse). Two weeks after the last injection, three mice from each group were sacrificed. Then the sera and splenocytes were used for evaluation of the cellular immune responses.

### ELISA

Enzyme linked immunosorbent assay (ELISA) was performed to detect the absolute quantity of cytokines. Blood samples were collected fourteen days after the third immunization from the mice and were allowed to clot for two hours at room temperature before centrifugation for 20 min at 1000 × g. Sera were then isolated from the clot and stored at −20 °C or −80 °C until used. The concentrations of IL-4 and IFN-γ in the sera were determined by commercial ELISA kits according to the manufacturer’s instructions (eBioscience, San Diego, CA, USA). All assays were performed in triplicate.

### *In vitro* CTL activity

Fourteen days after the last vaccination, mice were sacrificed and splenocytes harvested by ammonium chloride lysis buffer (NH_4_Cl, 100 mM and Tris 0.2 M). Viable splenocytes were counted using trypan blue (0.4%, w/v) and the cells were re-stimulated with the 10^8^ PFU GP2 displaying phages (100 μL). Target cells (TUBO) were incubated with Calcein AM (12.5 µM, Calcein-AM, Invitrogen, USA) at 37 °C in the dark for one hour^31^. To the maximum and minimum release wells Triton X-100 (2%) and culture medium were added respectively. Using a fluorescent plate reader (FLX 800, BioTek Inc. USA), the fluorescence intensity was measured at 485 nm (excitation) and of 538 nm (emission). The specific lysis (percentage) was calculated using the formula: (release by CTLs - release by targets alone)/(release by 2% Triton X-100 − release by targets alone) ×100. CT26 cells (non-expressing rHER2/neu) were used as negative controls to indicate the specificity of cytotoxic activities.

### INF-γ and IL-4 cytokines mRNA expression

To evaluate INF-γ and IL-4 cytokines mRNA expression in splenocytes isolated from spleen of immunized mice was used for Real-time Reverse Transcription-PCR (RT-PCR) assay. Using high pure RNA tissue kit (Roche, Germany) total RNA was extracted from homogenized spleen tissue (based on instructions provided by the manufacturer). A Nano Drop spectrophotometer (ND-1000) was used to quantify the extracted RNA and the samples stored at −80 °C until use. A 100 ng total RNA was used in real time RT-PCR in a one-step SYBR Green real time RT-PCR (Invitrogen, California, USA). For one-step real time RT-PCR amplification and SYBR Green fluorescence detection, the Applied Biosystems StepOne Real-time PCR System (Life Technologies Corporation, Carlsbad, CA) was used. The following three pairs of primers were used separately: one pair for the endogenous housekeeping control gene β-actin (F: TGACCGGCTTGTATGCTATC and R: CAGTGTGAGCCAGGATATAG) and two pairs to amplify INF-γ (F: GCTCTGAGACAATGAACGCT; R: AAAGAGATAATCTGGCTCTGC), and IL-4 (F: TCGGCATTTTGAACGAGGTC; R: GAAAAGCCCGAAAGAGTCTC^32,33^. To confirm the specificity of primers and possible contamination, a negative control was included in each run. Melt curve analysis was used to assess the possibility of nonspecific amplification or primer-dimmer formation. To evaluate fold changes of mRNA levels in the immunized group compared to control group, the comparative CT (threshold cycle) method was employed. The Step One System software was used to calculate fluorescence CT. The levels of mRNA were normalized to the endogenous reference gene β-actin (∆CT) and then relative to a control group (ΔΔCT), subsequently fold changes were expressed as “log _2_ [2(−∆∆CT)]”. The average valuse were calculated from three runs per sample.

### *In vivo* studies for prophylactic effects

Two weeks after the last booster, TUBO cells (5 × 10^5^ cells in 50 µL PBS buffer) were injected subcutaneously (SC) in the right flank of immunized BALB/c mice (seven mice in each group). Mice were monitored every day. A digital caliper was used to measure three orthogonal diameters of the developing tumor (a, b, c). The tumor volumes were calculated based on the formula [(height × width × length) × 0.5]. Time to reach the end point (TTE) was calculated based on the equation of the line obtained by exponential regression of the tumor growth curve. The difference between the median TTE of treatment group (T) and the median TTE of the control group (C) were used to calculate the percent TGD (the percent of tumor growth delay) (TGD % = [(T − C)/C] × 100]) for each mouse^34^. To follow the ethical committee rules, mice were sacrificed if the following conditions observed; the body weight loss was over 15% of the initial weight, the tumor volume was greater than 1000 mm^3^ or the mice became sick and unable to feed.

### *In vivo* therapeutic effects

To evaluate the anti-tumor efficacy of GP2 displaying phages and control λF7, 5 × 10^5^ TUBO cells (in 50 µL PBS buffer) were injected subcutaneously in the right flank of four to six week old female BALB/c mice. Fourteen days after tumor inoculation, 10^8^ PFU of GP2 displaying phages and λ F7 (100 µL/mouse) were injected subcutaneously for three times at two week intervals. The λ F7 and TN buffer were used for control groups. Mice were monitored regularly every day. The tumor volume was measured and calculated as mentioned in the above section.

### Statistical analysis

To assess the significance of the difference among various formulations, Descriptive statistics, the one-way analysis of variance (ANOVA) and Tukey test, Independent T-test and Log-rank test for survival analysis were used (Graph Pad Prism Software, version 6, San Diego, CA). The *P* value < 0.05 was considered to be statistically significant.

## Results

### High levels of INF-γ in gpD::GP2 group

**Figure 1:**
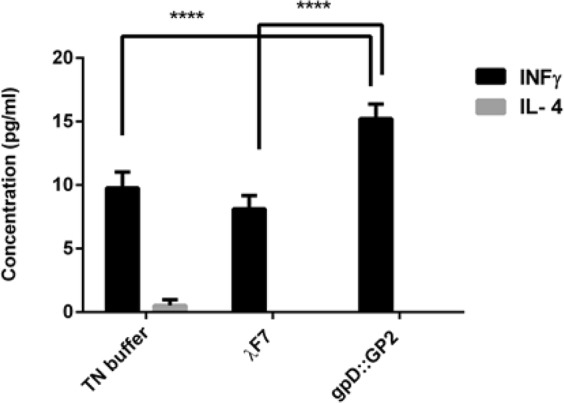
ELISA results for cytokine production by gpD::GP2 displaying vaccinated mice. Animals were vaccinated with three subcutaneous injections of 10^8^ PFU gpD::GP2 every two weeks. Negative controls included 10^8^ PFU of λF7 or TN buffer. Two weeks after the last booster, sera were taken from three mice for each group and the concentrations of cytokines were measured using IFN-γ and IL-4 ELISA assay kits. Data are shown as mean ± SEM. (n = 3). ****P < 0.0001; denotes significant difference compared to controls.

To evaluate the induction of anti-tumor T-cell response, the sera from the immunized mice was collected two weeks after the last injection. ELISA assay indicated that the gpD::GP2 treated mice in comparison to other groups, produced significant secretion of IFN-γ (*P* < 0.0001). None of the gpD::GP2 and λ F7 groups exhibited a sizable IL-4 response in mice (Fig. 1).

### Antigen-specific cytotoxicity by gpD::GP2

**Figure 2:**
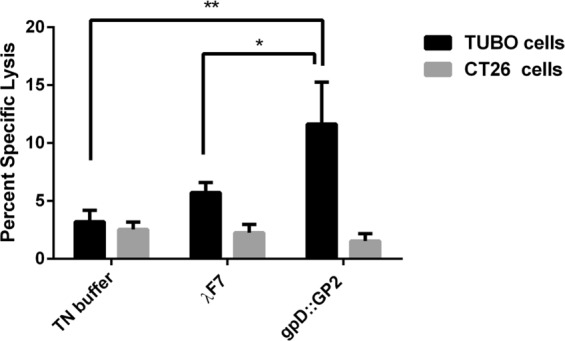
*In vitro* cytotoxicity of splenocytes (antigen-specific CTL response) isolated from immunized mice. This response was measured using Calcein AM-loaded rHER2/neu-expressing TUBO cells and compared to non rHER2/neu expressing CT26 cells. Data are shown as mean ± SEM (n = 3). *P < 0.05 and **P < 0.01; denotes significant difference from controls.

To determine the lytic activity of T cells against tumors, cytotoxicity assays would provide an *in vitro* evaluation of the response^35^. The gpD::GP2 phage exhibited a significantly effective response in generating CTL cytotoxicity. The vaccine reacted with the TUBO cells expressing rHER2/neu in comparison with the TN buffer (*P* < *0.01*) and λ F7 groups (*P* < 0.05). This cytotoxic activity was antigen specific because the CTL response was not seen against the rHER2/neu negative CT26 tumor cells (Fig. 2).

### High expression of IFN-γ in the gpD::GP2 group

Considering the expression of IL-4 and IFN-γ, the gpD::GP2 vaccine group exhibited the highest levels of CTL cytotoxic activity in the immunized mice as compared to the controls. This trend was also supported by the real time RT- PCR analysis. It was shown that the gpD::GP2 vaccine group modulated mRNA expression of both cytokines IL-4 and IFN-γ in favor of an efficient CTL cytotoxic responses.

**Figure 3:**
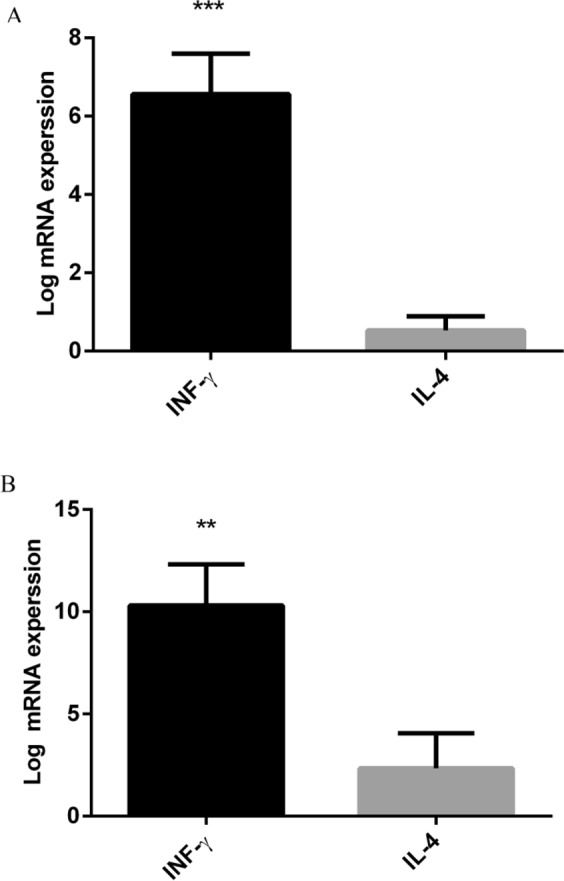
Higher IFN-γ and IL-4 expressions of in splenocytes isolated from BALB/c mice immunized with gpD::GP2 fourteen days after the final vaccination with 10^8^ PFU of gpD::GP2 phages compared to control. (**A**) Log-fold changes in gene expression compared with the TN buffer are expressed. (**B**) Log-fold changes in gene expression compared with λ F7 are expressed. β-actin was used to normalize gene expression levels for each sample. All values show means ± SD (n = 3). ***P < 0.001 and **P < 0.01; denotes significant difference compared to controls.

The assays showed that IFN-γ secretion was increased by 6.56 ± 1 (*P* < 0.001) in mice immunized with gpD::GP2 splenocytes compared to the TN buffer group two weeks after the last immunization, whereas log of fold changes in IL-4 expression assay was decreased significantly (Fig. 3A). In the gpD::GP2 group the expression of IFN-γ and IL-4 was compared with those of the λ F7 group. It was demonstrated that in the gpD::GP2 group IFN-γ expression was increased significantly (*P* < 0.01) while the expression of IL-4 decreased compared to the controls (Fig. 3B).

### Prophylactic study

**Figure 4:**
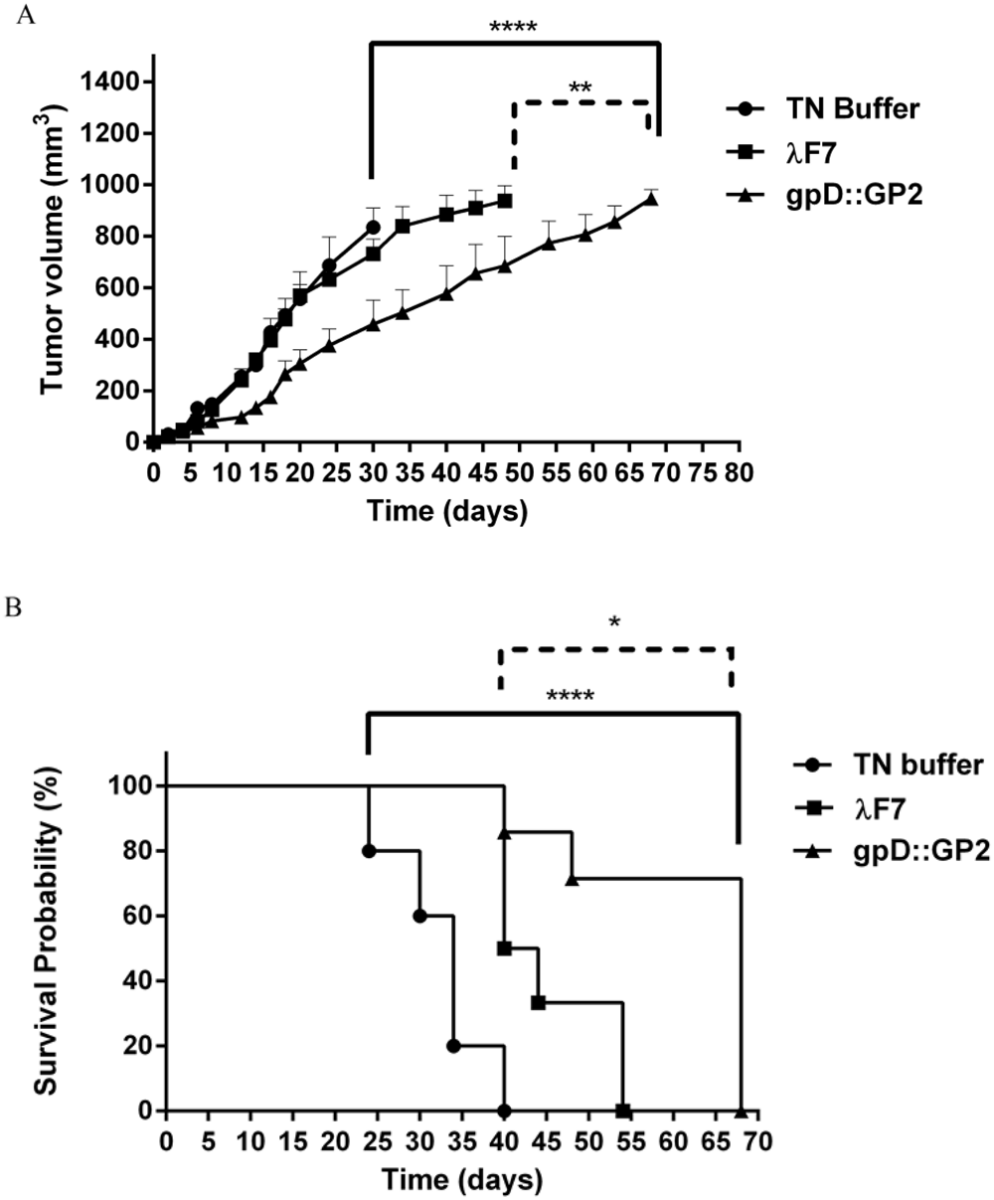
A representation of the results obtained in prophylactic assays. The protective immunization of BALB/c mice with gpD::GP2 phages against a TUBO cell implantable tumor model. Fourteen days after the last vaccination, seven mice in each treatment group were challenged with 5 × 10^5^ TUBO cells subcutaneously. Mice were monitored for tumor growth (**A**) and survival (**B**). The tumor size was calculated twice per week. Mice monitoring was conducted for 70 days. The data indicate mean ± SEM (n = 7). *P < 0.05, **P < 0.01, ***P < 0.001 and ****P < 0.0001; denotes significant difference from the control groups.

The analysis of tumor growth curve indicated that the gpD::GP2 group proved to be prophylactically effective as it significantly reduced the growth rate of the tumor (*P* < 0.0001) compared with TN buffer and the λF7 group (*P* < 0.01) (Fig. 4A). The prophylactic effects observed in mice groups are presented in Table 1 which indicates median survival time (MST), time to reach end point (TTE) and tumor growth delay (% TGD) for all treatment groups.

**Table 1:** Comparative presentation of the cancer prophylactic effects of different vaccine groups.

Group	MST^a^ (day)	TTE^b^ (day ± SD)	TGD^c^ %
TN buffer	35	32 ± 5.4	—
λ F7	42	48 ± 13.7	51
gpD::GP2	68	61 ± 13.0	89^****^

^a^Median survival time.

^b^Time to reach end point.

^c^Tumor growth delay.

^****^Denotes significant difference compared to other groups. (n = 7).

Survival analysis (up to two months, 60 days) revealed that the gpD::GP2 vaccine group significantly prolonged MST, TTE and % TGD compared to the TN buffer treated (*P* < 0.0001) and the λF7 treated groups (*P* < 0.05) (Fig. 4B).

### Therapeutic activity study

**Figure 5:**
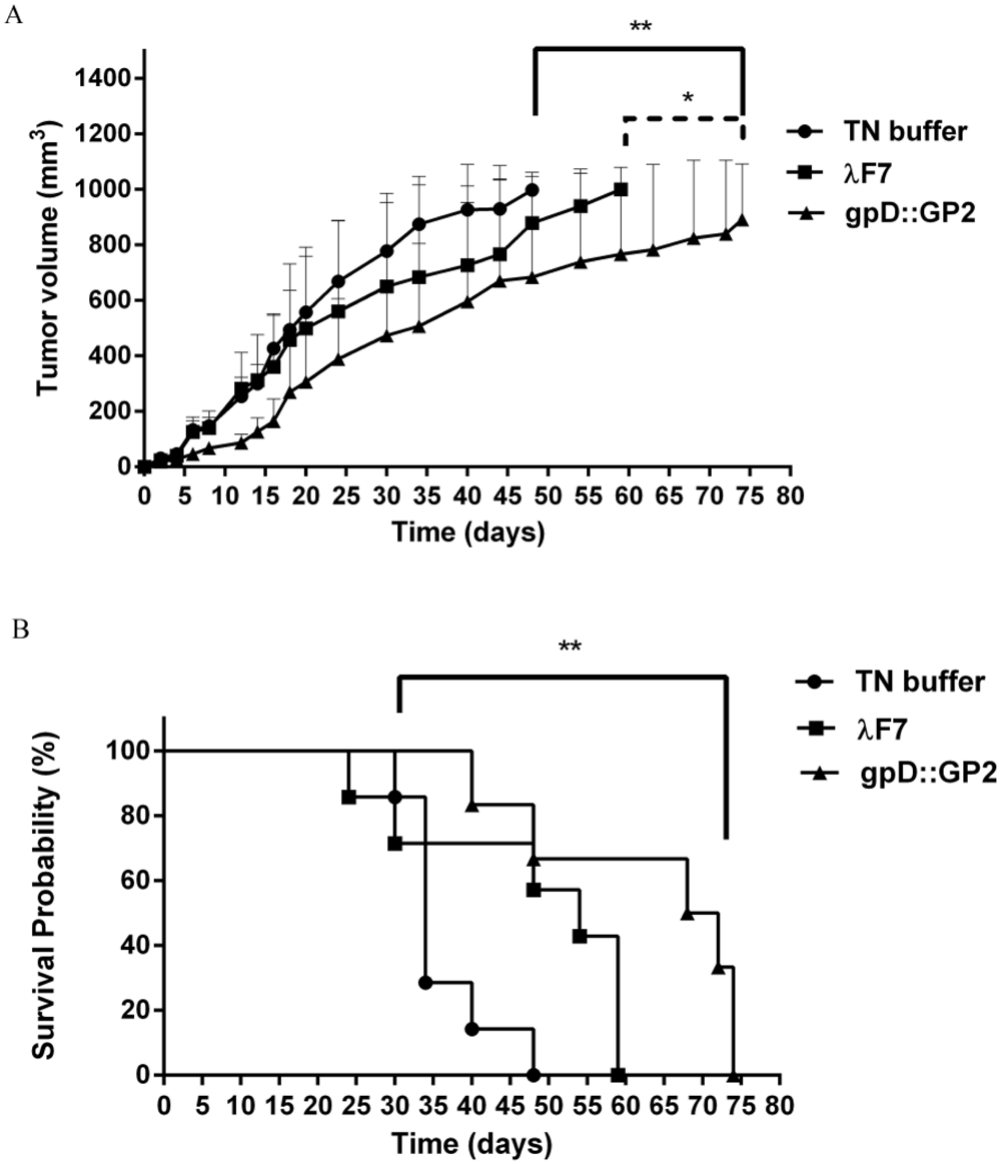
Therapeutic effects of vaccination with gpD::GP2 displaying phage nanoparticles against the BALB/c mice TUBO tumor. Fourteen days after the tumor induction the mice were subcutaneously injected with chimeric phage test and controls for three times at two week interval. The tumor size was calculated based on the measurement of three dimensions. (**A**) Tumor growth was measured twice per week. (**B**) Mice survival was followed for 91 days. The data indicate mean ± SEM (n = 7). *P < 0.05 and **P < 0.01; denotes significant effects compared to the TN buffer group.

Based on the considerable T-cell response in immunized mice, the tumor therapeutic activity of the chimeric phage in TUBO tumor model of BALB/c mice was evaluated. Among different mice groups, the gpD::GP2 group was more efficient in inhibition of tumor growth rate compared to the TN buffer (*P* < 0.01) and λF7 groups (*P* < 0.05) (Fig. 5A). The therapeutic efficacies of the groups are presented in Table 2. The data indicates the median survival time (MST), time to reach end point (TTE) and tumor growth delay (TGD %) for each mice group. The survival analysis results represented in a Kaplan-Meier plot were used to analyze significant differences in therapeutic efficacy between the control groups. Mice treated with gpD::GP2 showed the longest MST, TTE and % TGD in comparison to the TN buffer group (*P* < 0.01).

**Table 2:** Therapeutic efficacy data against the TUBO tumor model of mice.

Group	MST^a^ (day)	TTE^b^ (day ± SD)	TGD^c^ %
TN buffer	34	38 ± 8.3	—
*λ* F7	54	46 ± 15.9	21
gpD::GP2	70	58 ± 17.1	53^**^

^a^Median survival time.

^b^Time to reach end point.

^c^Tumor growth delay.

^**^Denotes significant difference from all other formulations (n = 7).

## Discussion

**Figure 6:**
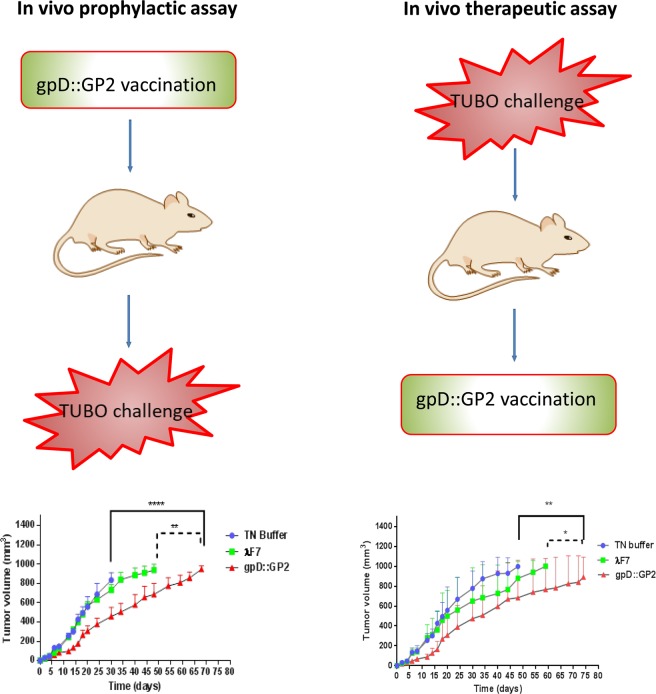
A schematic summary of the breast cancer vaccine study (gpD::GP2) in a TUBO tumor model of BALB/c mice.

This study aimed to assess the immunologic stimulation of a λ phage construct displaying the GP2 peptide derived from the HER2/neu proto-oncogen. The phage construct was surface decorated with its gpD coat protein fused to the GP2 immunogenic peptide. Furthermore, we planned to test the effectiveness of λ nanoparticles as safe human adjuvants to compensate for the reduced protein copy numbers seen in other immunogenic peptide settings. The phage was expected to reduce the dose needed for an effective response and to improve the immunogenicity and protective potential of the formulation. In recent years, phage nanoparticles displaying peptides derived from tumor antigens have attracted great interest as cancer vaccine delivery systems. This is due to the phage vector advantages including its intrinsic adjuvant activity, high multivalent display potential, safety profile, and its ease of manufacturing and construction. The displayed peptides on the phage particles are accessible and can elicit immune responses in different animal systems^36^. Peptides alone have a poor immunogenic profile as well as short life-spans; both intracellular and in serum^37^. Soluble antigens cannot enter the appropriate intracellular compartment to undergo processing and presentation on class I MHC molecules^38^. Recent studies revealed that patients vaccinated with GP2+ GM-CSF presented with a considerable reduction (37%) in the recurrence of cancer compared to untreated patients, and those patients that received GM-CSF alone presented with a 57% lower risk of cancer recurrence. Also, the primary vaccination of patients with GP2+ GM-CSF included six injections and boosters given every three to four weeks which involves a procedure taking a high time and cost^39^. The patients experienced grade one systemic and local toxicity which was due to the GM-CSF including erythema, headache, pruritus, fatigue, bone pain, myalgia and flu-like conditions^40^. GM-CSF can cause an antitumor immune response; however, it can promotes cancer cell migration and proliferation in different types of cancers including lung cancer, skin carcinoma, and gliomas^41^. Suitable delivery systems based on immune-stimulating complexes that present with a long circulation time and also have tendency to be taken up more efficiently by APCs to induce CTL response can therefore provide considerable improvement in vaccination^42^. Recently, bacteriophages have been sought after as an attractive alternative in novel vaccine research, particularly as delivery platforms for peptide and protein-based vaccines against infectious diseases and cancer^43,44^. The efficacy of bacteriophage-driven APC delivery and cross-presentation has been proven in comparison to free antigens. This increase in efficacy includes greater cellular uptake, higher immunogenicity levels in addition to the lower costs seen in bacteriophage production^45^. Bacteriophages are endogenous adjuvants, aiding in the direction of the immune response^46^. In different research settings, M13 and f1 (filamentous phages) have been used for bacteriophage deliveries^47^. However, phage λ is not only capable of displaying large proteins via gpD fusions, but it can also tolerate a density where approximately 90% of the incorporated D protein is a fusion^48^. In a temperate phage the phage protein development is repressed in the phage lysogenic state and occurs just before the cell lysis, so toxic display peptides will have less influence on the cell activity needed during phage production^49^. The λ library displayed a 100-fold higher display for all fragments compared to filamentous phage when tested using in an antibody binding assay. Overall, the λ system was able to display proteins of different sizes, with the number of fusions displayed on each phage particle being 2–3 orders of magnitude greater than that of M13^50^. Functional proteins such as λ -lactamase, luciferase (a 61 kDa protein), or even λ -galactosidase (a 465 kDa protein), have all been displayed on λ with no poor effects on viability and morphology^51^. Phage therapeutic endeavors also have included the use of a T7 and λ for phage display to identify antigens eliciting a B cell response in cancer^52^. One study has shown that a hybrid M13 phage displaying epitope LKVIRK in the N-terminal region of the major coat protein (pVIII) could induce high levels of IFN-ɣ in the CD4^+^ splenocytes during a one week post-inoculation in C57BL/6 mice^53^. Filamentous phage particles containing the expression cassette of Herpes Simplex Virus 1 (HSV-1) glycoprotein D could induce humoral and cellular immune responses in BALB/c mice^54^. Clark and March (2006) showed that recombinant λ phage particles containing expression cassette of hepatitis B surface antigen (HBsAg) could induce specific antibodies in mice and rabbits^55^. Murine pneumotropic virus (MPtV) or murine polyomavirus (MPyV) VLPs carrying an ECD-TM (extracellular plus trans membrane domain) fragment of rHER-2/neu have proven efficacy as prophylactic and therapeutic tumor vaccines against rat HER2-positive TUBO tumors^56^. Thomas *et al*. (2012), reported that hybrid DNA and peptide inoculant λ gfp10-GFP-TAT could stimulate the most specific and greatest amplitude of an IFN-γ production in female CD1 mice^57^. In the current study, we employed λF7 phage particles displaying the GP2 peptide as a fusion to the gpD coat protein of the phage (gpD::GP2) fusion. The immunogenicity and antitumor potential of the bacteriophage nanoparticles displaying the GP2 peptide derived from HER2/neu were investigated using *in vivo* and *in vitro* assays. A schematic model diagram of our study is presented in Fig. 6. BALB/c mice were immunized subcutaneously three times with 10^8^ PFU of endotoxin-free gpD::GP2 phage nanoparticles and the phage λF7 and TN buffer were selected as controls. Our data demonstrated the *λ* phage activity as an endogenous adjuvant. This was observed by significant IFN-γ splenocyte proliferation in the absence of an adjuvant in our inoculations. The adjuvant activity is likely linked to the *λ* capsid, or from bacterial pathogen-associated molecular patterns (PAMPs). Fourteen days following the last booster injection, splenocytes and sera were extracted from spleens and blood. ELISA showed that mice immunized with gpD::GP2 nanoparticles could induce a significantly higher INF-γ and CTL response compared control groups indicating the crucial role of repetitive display of GP2 peptide on the surface of λ nanoparticles in its immunogenicity. To demonstrate that immunization with gpD::GP2 phage nanoparticles induces peptide-specific CTLs capable of killing GP2 peptide-pulsed target cells *in vitro*; as expected from IFN-γ ELISA results, compared to controls, a highly significant lysis of target cells was observed in the mice vaccinated with gpD::GP2 nanoparticles. The CTL response was shown to be associated with lower and higher amounts of IL-4 and IFN-γ respectively, in mRNA expression and protein assays. The CTL responses elicited by vaccination of mice with the gpD::GP2 nanoparticles exhibited a latency in tumor growth and presnetd with superior anti-tumor effects *in vivo*. It was indicated by a higher survival (89% vs. 51%) of mice against HER2-over expressing TUBO cell line challenge. In contrast, no protection against TUBO cell challenge was observed in control animals inoculated with TN buffer and all mice developed fast-growing tumors and had to be euthanized much earlier than the chimeric phage treated animals. In accordance with *in vivo* results obtained in IFN-γ, ELISA and cytotoxicity assays, immunization of mice with gpD::GP2 nanoparticles could prevent the TUBO tumor development in mice. Also the therapeutic assay showed that gpD::GP2 nanoparticles could induce a complete regression of the established tumors (53% vs. 21%). We report that the designing of a linker (GG or GS) helps correct cross presentation of polytopes to the immune system. Yeast-derived Ty-VLPs carrying two different CTL epitopes linked by a glycine-glycine (GG) or glycine-serine (GS) spacer successfully evoked T cell responses against both epitopes^58^. Interestingly, only the gpD::GP2 chimera in which the GP2 peptide was directly linked to gpD by an in-frame short linker contains GG and GS (T-S-G-S-G-S-G-S-G-S-G-S-G-S-G) flexible space was successfully processed and cross-presented and induced effective anti-tumor CTL responses. The data supports the previous reports that glycine and serine flanking residues enhance cross-presentation of Yeast-derived Ty-VLPs carried CTL epitopes. Our results indicate that the λF7 test construct decorated with the fused peptide gpD::GP2 successfully decreased the tumor size in prophylactic (*P* < 0.01) and therapeutic (*P* < 0.05) assays in comparison to the TN buffer. Also, we have seen longer survival time only in prophylactic assay (*P* < 0.5). The control λF7 did not show *in vitro* induction of INF-γ and CTL activity compared to the gpD::GP2 group. Finally, the results indicated that the *in vivo* anti-tumor efficacy of the phage delivery system was mediated by the induction of tumor protection in BALB/c mice vaccinated with gpD::GP2 nanoparticles. Here, we report the successful *in vivo* and *in vitro* induction of INF-γ and CTL activity immune responses in mice vaccinated with λF7 (gpD::GP2). It is noteworthy to mention that bacteriophages are endogenous adjuvants and it seems that they can stimulate INFɣ from CD4+ cells. Moreover, they are considerably safe in mammalian systems and their efficacy has been shown in humans, despite the host induced production of anti-bacteriophage Abs^59^. Additional studies including the evaluation of tumor microenvironment conditions and its interaction with the immunogenic phage constructs would be an interesting subject of the future research.

## Conclusions

In conclusion, we demonstrated that compared to the control groups, the delivery of GP2 peptide displayed on a non-pathogenic λ bacteriophage, significantly enhanced the anti-tumor immune function in BALB/c TUBO mice model. We think that the observed immunogenicity of the gpD::GP2 phage nanoparticles as both protective and inhibitors of tumor against HER2/neu overexpressing implantable tumor deserves more investigation.

## Data Availability

Raw data were generated at Biotechnology Research Center, Mashhad University of Medical Sciences. Derived data supporting the findings of this study are available from the corresponding author JB on request.
